# Bioactivity and Chemical Synthesis of Caffeic Acid Phenethyl Ester and Its Derivatives

**DOI:** 10.3390/molecules191016458

**Published:** 2014-10-13

**Authors:** Pengxuan Zhang, Yuping Tang, Nian-Guang Li, Yue Zhu, Jin-Ao Duan

**Affiliations:** 1 Jiangsu Collaborative Innovation Center of Chinese Medicinal Resources Industrialization, Nanjing University of Chinese Medicine, Nanjing 210023, China; 2 Jiangsu Key Laboratory for High Technology Research of TCM Formulae, Nanjing University of Chinese Medicine, Nanjing 210023, China; 3 National and Local Collaborative Engineering Center of Chinese Medicinal Resources Industrialization and Formulae Innovative Medicine, Nanjing University of Chinese Medicine, Nanjing 210023, China

**Keywords:** caffeic acid phenethyl ester, chemical structure, biological, synthetic method, structural modification

## Abstract

Caffeic acid phenethyl ester (CAPE), as one of the main active ingredients of the natural product propolis, shows the unique biological activities such as anti-tumor, anti-oxidation, anti-inflammatory, immune regulation, and so on. These have attracted the attention of many researchers to explore the compound with potent biological activities. This review aims to summarize its bioactivities, synthetic methods and derivatives, which will be helpful for further study and development of CAPE and its derivatives.

## 1. Introduction

**Figure 1:**
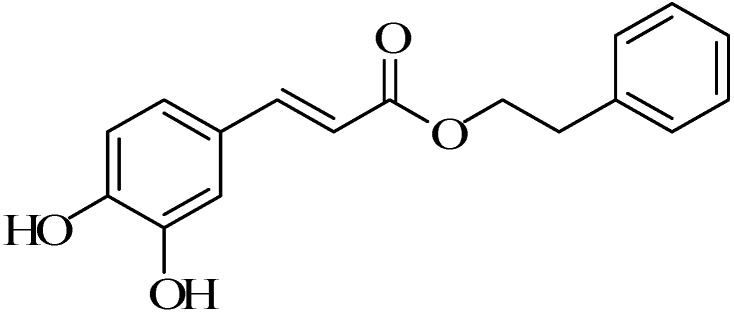
Chemical structure ofcaffeic acid phenethyl ester (CAPE).

Caffeic acid phenethyl ester (CAPE) (Figure 1) was acquired from propolis obtained through extraction from honeybee hives [1], which contained more than 180 compounds. The chemical compositions of propolis from different areas were different, as well as the CAPE content. The CAPE content from China propolis was highest, up to 15–29 mg∙g^−1^, but from Brazil, propolis basically did not contain CAPE [2]. However, CAPE showed an important role in the bioactivities of anti-tumor [3,4], anti-oxidation [5,6], anti-inflammatory [7,8], immune regulation [9] and so on. With the study of the bioactivities of CAPE, the research and development of new drugs were in urgently development. However, the scale preparation of raw materials was the basic bottleneck in its industrialization. So, the large-scale preparation of CAPE had been the hotspot and difficulty. Therefore, this paper summarizes research progress about the synthesis of CAPE and its derivatives.

## 2. Biological Activities

### 2.1. Anti-Oxidant and Anti-Inflammatory Activities of CAPE

The structure of CAPE contained catechol which was a strong antioxidant [9]. Through the study of the antioxidant activity of the extraction of propolis on acute renal injury in rats, it was shown that the antioxidant activity of the extraction was better than VE, and CAPE was one of the main components [10].

Modern medical research showed that the main antioxidant groups of propolis were CAPE, caffeic acid, quercetin, kaempferol, galangal and cinnamic acid ester. The antioxidant effect of propolis which contained CAPE was stronger than that without CAPE, and the antioxidant effect of CAPE was stronger than that of galanga, which proved that CAPE played an important role in the antioxidant activity [6].

Propolis has been for a long time used to treat burns. Burns led to insufficient blood volume, ischemia, and body damage caused by ischemic reperfusion. In addition, intestinal mucosal damage burns could also cause endotoxemia. These changes appeared in the form of chain reactions, such as the free metal ion (iron) released from the metabolic process of the arachidonic acid (AA), leading to the generation of hydroxyl radicals by Fenton reaction with perhydrol, the release of inflammatory cytokines, the aggregation of platelets and changes of other hormone metabolism. These reactions were triggered by the oxidation reaction, leading to reactive oxygen species’ (ROS) excessive production and release. Oxidant changes of local and systemic could stimulate tissue inflammation and weight gain, resulting in the blockage of neutrophils and macrophages in other tissues and preventing the improvement of the inflammation. Accordingly, the antioxidant system tissue was completely destroyed, and was unable to cope with ROS at the late stage of the offensive. The increase of oxidant and the decrease of endogenous enzymatic or the non-enzymatic antioxidant activity would cause lipid peroxidation of cell membranes and cell energy attenuation. Lipid peroxidation of cell membrane could lead to the changing of the liquidity and permeability of cell membranes, and increase the degradation rate of protein and nucleic acid, which eventually led to cell lyses. Therefore, the harmful effects of burns were not only limited to local skin damage, but extended to other organs as well. After burning, the activities of MDA, myeloperoxidase (MPO) and catalase (CAT) in cells increased obviously, superoxide dismutase (SOD) and xanthine oxidase (XO) activities decreased too. In 2004, A. Gurel tested whether CAPE had the activity to treat the oxidative damage from burns by Wistar albino rats. They used boiling water (100 °C) to burn 25%–30% of the body surface of rats, intraperitoneally injected 10 μmol∙kg^−1^ of CAPE into burned rats daily, and 1 day, 3 days, 7 days after burning injury into each organ, studied the impact on lipid peroxide in serum. Results showed that CAPE could obviously reduce MDA, MPO and CAT levels of kidneys and lungs, and increase the activities of SOD and XO for burned rats [11].

In addition, Michaluart and Mirzoeva reported CAPE could inhibit the synthesis of prostaglandin (PG) and leukotriene [12]. PG was produced by AA under the catalysis of COX. COX had two isomeric forms, which were COX-1 and COX-2, and PG played an important role in the progress of the formation of the inflammatory. CAPE had the antioxidant activity by inhibiting the release of AA, and the activities of COX-1 and COX-2.

Studying the oxidation in heart tissue of rats with diabetes induced by streptozotocin (STZ), Okutan found CAPE had a great impact on lipid peroxidation in diabetic rat heart and the activity of antioxidant enzyme could significantly reduce SOD in diabetic rat heart tissue and the content of CAT [13]. As a result, CAPE obviously displayed antioxidant activity in the study. Furthermore, Pan reported [14] the protective effect of caffeic acid phenethyl ester on myocardial injury due to antioxidant action. Kim proposed that the indirect antioxidant activity of CAPE against oxidative stress in HepG2 cells was partially attributed to induction of HO-1, which was regulated by Kelch-like erythroid-cell-derived protein with CNC homology (ECH)-associated protein 1 (Keap 1)-independent Nrf2 activation relying on post-translation phosphorylation of ERK [15].

Yang reported that CAPE inhibited the expression of IL-6, MCP-1 and ICAM-1 induced by the pro-inflammatory cytokine IL-1β in corneal fibroblasts. The activation of AKT and NF-κB by IL-1β was markedly inhibited by CAPE, whereas the activity of mitogen-activated protein kinases (MAPKs) was not affected. CAPE significantly suppressed the IL-1β-induced migration of differentiated HL-60 and THP-1 cells [16].

Cho reported CAPE promoted anti-inflammatory effects by inhibiting MAPKs and NF-κB signaling in activated HMC-1 human mast cells [17].

### 2.2. Immunomodulatory Activity of CAPE

CAPE in propolis with immune regulating activity could change the cell structure of the inflammatory body of thymus and spleens, and reduce the weight of thymus at the same time. CAPE also could directly or indirectly reduce the number of T cells. The immunosuppressive behavior of CAPE had been evaluated in T-cells [18], because the causative agent for inflammation was T-cells in general [19]. In Jurkat cells, binding with DNA and the activities of transcription factors, such as NF-κB, nuclear factor of activated cells (NFAT), and activator protein-1 (AP-1), were also characterized to examine the mode of inhibition of the transcription phase by CAPE [20,21,22]. The result showed the CAPE-mediated inhibition of NF-κB-dependent transcription had no effect on the disposition of IBκB. Chen reported CAPE could induce apoptosis of human leukemia HL-60 cells, reduce the concentration of granulocytes of neutrophil and white blood cells and monocytes from inflammatory sites, and the concentration of tissue fluid exudation [23].

In addition, CAPE could inhibit the production of NO, which was a free radical produced by iNOS catalyzing L-arginine. Too much NO could cause damage to the body. The report showed that CAPE inhibited the generation of NO of mouse peritoneal macrophages induced by Lipopolysaccharide (LPS) [24]. Kageura conducted experiments with mice that had never been produced, measuring the IC_50_ of the NO in on mouse peritoneal macrophages induced by LPS, which was 4 μg∙mL^−1^ [25]. However, Matsuda conducted a verification experiment, finding that the IC_50_ was 15 μg∙mL^−1^ [26]. Yun reported that CAPE could inhibit the content of NO induced by LPS + IFN-γ, which was mainly due to CAPE acting on NF-κB sites in the iNOS promoter, and directly inhibited the catalytic activity of iNOS inhibiting the transcription of iNOS gene. Therefore, the CAPE could decrease the expression of NOS gene to immune regulation [27].

Because virus resistance is ever increasing, the number of antiviral drugs used for AIDS patients who have already been deemed invalid is too many to enumerate examples. Although mankind had been unremittingly fighting against AIDS, incidence of AIDS in recent years had continued to rise because of the human immunodeficiency virus (HIV). Progress in AIDS treatment lagged far behind the pace of HIV infection, while frequent occurrence of the virus resistant phenomenon made it more difficult for the effective treatment of AIDS. It could be said, for AIDS treatment with drugs relative to the single market, innovative drug preparation effectively had become an increasing. How to fight the virus resistant to invasion had become the research focus of antiretroviral drugs. The formation of HIV integrase depended on the life cycle of the virus, but not the homologous human cells [28]. HIV was the use of the enzyme which integrated its own genetic material to the infected cells. Integrase inhibitors are additional, new experimental antiretroviral drugs, which target the integration of enzyme. Integrase inhibitors showed anti-HIV activity by causing the mutation of the integrase binding site *in vitro*, which had synergistic effect with nucleoside reverse transcriptase inhibitor (NRTIs), non-nucleoside reverse transcriptase inhibitors (NNRTIs) and protease inhibitors (Pis). CAPE, as an integral part of propolis, had shown immune regulating performance [29].

In the 20th century, CAPE had been found to inhibit the activity of human immunodeficiency virus type 1. At present, CAPE had been confirmed as the HIV-1 inhibitor [30]. Its mechanism might be the unique molecular structure of CAPE which inhibited the reaction involved by nuclear transcription factor NF-κB [31], and interrupted the method of the treatment for multiple growing points in the life cycle of HIV, overcoming the resistance caused by a single goal orienting antiviral treatment [32]. Changing the structure of CAPE, such as replacing benzene ring with naphthalene ring, the inhibition of integrase would disappear at the same time [28], which proved that the benzene ring of CAPE had a specific structure-activity relationship to the integrase. For this reason, the inhibition of HIV by CAPE had become an important research direction of the newly developed anti-AIDS therapy.

### 2.3. The Anticancer Activity of CAPE

Cancer is one of the world’s major risks to human health. Since the 1970s, China’s cancer incidence rate has been rising. It has been hoped that the use of active substances in natural products could change, or postpone carcinogenesis, and prevent cancer at the same time. Through the study of natural anticancer compounds, we could develop a new anticancer drug.

Epidemiological studies had shown that eating a variety of rich vegetables, fruit and other natural foods, could significantly reduce the risk of cancer in humans [33], partly because natural foods contained esters, such as CAPE or benzene ester, which could inhibit certain types of cancer cell growth and selectivity [34]. CAPE was subsequently considered to be a potent anticancer component of propolis [35], and it was a natural anticancer drug with a good curative effect, and with little side effect [36,37]. CAPE was an earlier confirmed antitumor component against general *in vivo* and *in vitro* neoplasm models, melanomas, lung and prostate cancers and so on [38,39].

Research showed that CAPE in propolis had great impact on melanoma, colorectal and gastric cancer cell lines [40]. Hye believed that CAPE played a potential role in inhibiting tumor invasion and metastasis. This role was decided by the regulation of matrix metalloproteinases (MMPs) in cells [41]. Cell MMPs was a kind of proteolytic enzyme dependent on metal ion, playing a key role in the process of proliferation in the extracellular matrix. The activity of MMPs was regulated by three factors, which were the level of gene transcription, inactive enzyme precursor by proteolysis and activation, and the activity of tissue inhibitor of metalloproteinases (TIMP). CAPE could restrain the expression of cancer gene, treating fibrosarcoma by using different doses of CAPE. We found that CAPE could significantly inhibit the expression of genes. CAPE handled two P53 tumor mutant cell lines which were NCI-H358 and SKOV-3. Lacking type P53 cells could result in caspase-3 and DNA fracturing. In addition, CAPE could regulate the expression of human glial blastoma cell surface antigen GBM-18. The mechanism of CAPE inhibiting tumor included induction of apoptosis. CAPE had the specific destruction on tumor cells. In 1988, the America scholar Grunberger used viral induced cells transformed as materials, and found CAPE with a concentration of 2 ug∙mL^−1^ could effectively inhibit the abnormal cell growth. However, for normal mouse cells, even if the concentration of CAPE increased five times (10 ug∙mL^−1^), it was not toxic [42]. CAPE exhibited inhibitory effects on the motility and invasion of C6 glioma cells when tested with scratch assay and Boyden chamber assay. Furthermore, CAPE induced the expression of nerve growth factor and p75 neurotrophin receptor which were involved in neuroal cell differentiation and inhibited the activity of MMPs and induced the expression of RhoB, a tumor suppressor showing CAPE as an agent that possessed antitumor progression potential [43].

In 1995, Chiao extensively explored the role of CAPE in the transformation of cells induced by virus, and found CAPE could induce abnormal cell apoptosis, but normal cells were not affected [44]. At present, it was acknowledged that the formation of intestinal mucosal aberrant crypt foci (ACF) was the key stage of colorectal carcinogenesis. CAPE could reduce the formation of ACF of mice induced by carcinogen azoxymethane and the formation of the tumor could selectively kill the 26-L5 cancer cell lines with high mobility in mouse colon [45]. The EC_50_ for CAPE to colon cancer 26-L5 cells was 0.15 μmol∙L^−1^, and to HeLa cells was 10.7 μmol∙L^−1^.

The effect of CAPE on cholangiocarcinoma (CCH) growth both *in vitro* and *in vivo* was also studied. It decreased the growth of a number of CCH cells but not of normal cholangiocytes. On the other hand, Bax expression was increased whereas Bcl-2 expression was decreased in cells treated with CAPE. In BALB/c nude mice implanted subcutaneously with MzChA-1 cells treated with daily CAPE for 77 days, tumor growth was decreased and tumor latency was increased twofold [46].

Chiang found CAPE could significantly inhibit the growth of colorectal tumors in a mouse xenograft model. The mechanisms of action included a modulation of PI3-K/Akt, AMPK and m-TOR signaling cascades both *in vitro* and *in vivo* [47].

Concerning various combined studies (both *in vivo* and *in vitro*), Chen found the effects of CAPE on tumor growth in relation to IL-1β signaling when examined in CE81T (human esophageal cancer cells) and CCL-241 cells (human normal intestine cell line) [48]. CAPE significantly reduced xenograft tumor growth.

The effects of CAPE in a breast cancer model, including tumor growth both *in vitro* and *in vivo* were examined and its effects on the cell cycle, apoptosis, and angiogenesis in the hormone receptor negative MDA-231 and hormone receptor positive MCF-7 breast cancer cell lines were analyzed [49]. CAPE inhibited MDA-231 and MCF-7 human breast cancer cell growth.

CAPE was found to cause dose-dependent breast cancer stem cell self-renewal inhibition and progenitor formation [50].

### 2.4. Other Biological Activities of CAPE

Apart from the anticancer and anti-inflammatory properties of CAPE, other biological activities have been reported in recent years [51]. CAPE could effectively reduce the area of myocardial infarction induced by ischemia reperfusion in rats, reduce the spinal injury of ischemic reperfusion in rabbits, inhibit the auto oxidation of beta mercaptoethanol to produce the formation of reactive oxygen anions (super oxide), and loosen the rat thoracic aorta due to the shrinkage of phenylephrine and potassium chloride. CAPE provided a dual mechanism on blood vessels, on the one hand, activated with NO release at low concentrations, and on the other hand, activated at high concentrations, blocking the exchange of intracellular Ca^2+^, which meant CAPE could effectively avoid the complications associated with artery resection operations of the chest and with abdominal aortic arteries. Recently, CAPE was found to have slight chronic effects on pancreatic damage and hepatotoxicity in rats [52]. However, there has been no report that CAPE is harmful to normal cells [53].

To sum up, CAPE had been identified as the main component in propolis, and its bioactivities were very extensive, which led to suggesting that CAPE could have broad application in food, medicine and other fields.

## 3. Chemical Synthetic Method

Since 1988, Grunberger [4] reported the extraction of CAPE from propolis. CAPE was first chemically synthesized at Columbia University in 1988 [38]. Its synthetic method, especially the chemical synthesis, began to receive widespread attention.

### 3.1. Using Caffeic Acid as a Substrate for Synthesis of CAPE

#### 3.1.1. Catalyst for Directly Catalysing the Production of CAPE from Caffeic Acid and Phenethyl Alcohol

**Figure 2:**
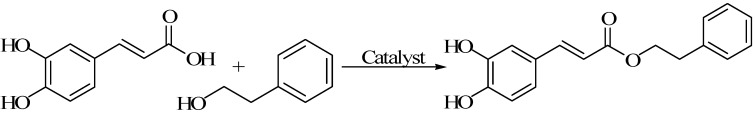
The scheme of direct catalysis of CAPE.

Caffeic acid and phenethyl alcohol was directly catalyzed for esterification for the synthesis of CAPE [54] (Figure 2).

The selection of catalyst on the synthesis of CAPE was very important, mainly in the following three situations: (1) The caffeic acid, SOCl_2_ and phenethyl alcohol were added in nitrobenzene solution, then mixed equimolarly, heated for 2 h, using the ether/hexane column to separate for CAPE. The yield was 60% [54]; (2) Toluene-p-sulfonic acid was used as the catalyst, benzene as dehydrating agent. Caffeic acid and phenethyl alcohol heated with the reflux reaction for 3–4 days, then the chromatography was used for separation of CAPE, the yield was 40% [55]; (3) The caffeic acid, dicyclohexylcarbodiimide (DCC) and dimethylaminopyridine (DMAP) were added to THF/CH_2_Cl_2_ (1:1) mixed solution, and stirred at room temperature. Then phenethyl alcohol was dropped slowly. TLC was used to track reaction. The ethyl ether/n-hexane column was used to obtain pure product, the yield was 46% [55].

The process of synthesis for directly catalysing the CAPE with caffeic acid and phenethyl alcohol and the selection of catalyst, had a great influence on the reaction rate of transformation. The transformation of traditional inorganic acid catalytic esterification had lower rates, but using SOCl_2_ as the catalyst for the conversion was higher.

#### 3.1.2. The Acyl Chloride Method

**Figure 3:**
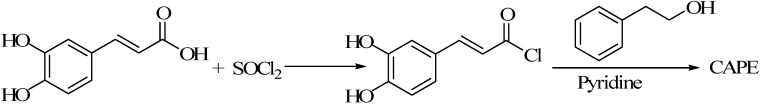
The scheme of method of acylhalide.

Caffeic acid with an excess of SOCl_2_ reacted at refluxing temperature about 1 h, then the unreacted SOCl_2_ was removed with vacuum distillation, getting solid intermediate which was acyl chloride. At room temperature, the mixed solution of phenethyl alcohol, pyridine and nitrobenzene were added, reacting for 1 h. The CH_2_Cl_2_/n-hexane column was used to obtain pure product, yield was 50%–86% [56] (Figure 3).

#### 3.1.3. The Reaction of Caffeic Acid and β-Phenyl Ethyl Bromide

**Figure 4:**
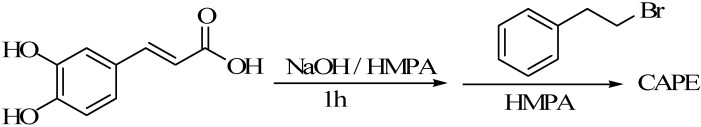
The scheme of halogen substituted hydrocarbon.

The reaction of caffeic acid and β-phenyl, of which the specific process was caffeic acid and sodium hydroxide, were dissolved into hexamethylphosphoramide (HMPA). 1 h after mixing, the mixed solution of β-phenyl ethyl bromide and HMPA was added. At room temperature, the ethyl ether/cyclohexane column was used to obtain pure product, yield was 70% (Figure 4).

Caffeic acid and potassium acid carbonate were dissolved into dimethyl sulfoxide, then the β-phenyl ethyl bromide was stirred in it, heated to 70 °C, reacted for 3 h, then cooled to 25 °C. Water was stirred into it to produce precipitations, and then the crude precipitations were obtained with filtering. The dilute potassium hydrogen carbonate and toluene solution was used to wash precipitations, and then the hot toluene used to cool and crystallize it to obtain pure product.

Caffeic acid and β-phenyl ethyl bromide could also generate CAPE in other kinds of solvent and catalyst systems. Because there were various kinds of system conversions, the reaction times were different. For example, using dimethyl sulfoxide and the NaOH with a mass fraction of 25% as solvent and catalyst gave a conversion rate of 30% in 50 h [55].

### 3.2. Using 3,4-Dihydroxy Benzaldehyde as a Substrate for Synthesis of CAPE

#### 3.2.1. Witting Reaction

Three phenyl phosphine acid phenethyl alcohol ester chloride and 3,4-dihydroxy benzaldehyde were dissolved into the mixed solution of CHCl_3_ and 1,4-dioxane. The K_2_CO_3_ was stirred into it. After ultrasound, TLC was used to track response. The reaction liquid was distillated. The n-hexane/ acetic ether column was used to obtain pure product; the yield was 71% [57,58].

**Figure 5:**
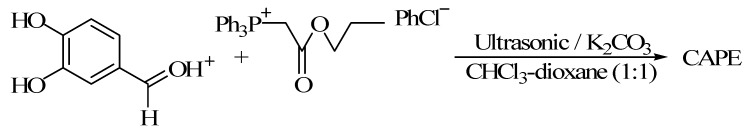
The scheme of witting reaction.

3,4-dihydroxy benzaldehyde and phosphonate ester could also generate CAPE under base catalysis [59] (Figure 5).

#### 3.2.2. Malonic Acid Monoester Method

**Figure 6:**
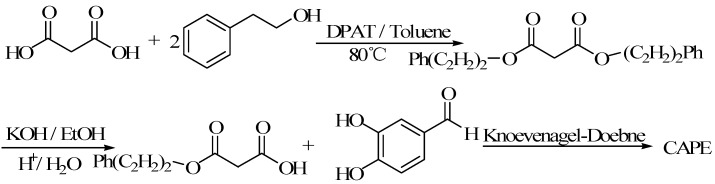
The scheme of method of malonic acid monoester.

Using toluene as solvent, catalyst DPAT catalysed malonic acid and esterified for malonic acid diester. It reacted equimolarly with KOH for malonic acid monoester potassium single. The malonic acid monoester was obtained by acidification, then condensated for CAPE with 3,4-dihydroxy benzaldehyde by Knoevenagel-Doebner [60] (Figure 6).

#### 3.2.3. One Pot Method

**Figure 7:**
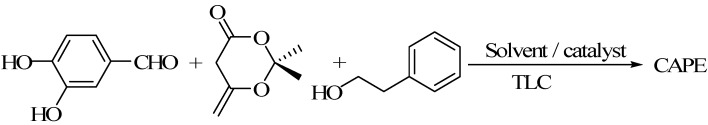
The scheme of method of one pot.

Using toluene as solvent, isopropylidene malonate and phenethyl alcohol reacted. After a period of time, 3,4-dihydroxy benzaldehyde and pyridine were added, stirred until dissolved. Then, piperidine was added. TLC was used to track response. The solvent was eliminated after reacting. The ethyl ether was added after the residual liquid cooling, stirred until dissolved. Dilute hydrochloric acid and water were used to wash it. The ethyl ether layer was collected, dried by anhydrous MgSO_4_ overnight. After filtrating, ethyl ether was evaporated. The toluene was used to recrystallize it to obtain pure products, yield was 68.9% [61] (Figure 7).

To sum up, the conditions of caffeic acid and phenethyl alcohol directly catalysing esterification for CAPE were simple, but time consuming, giving low yield. Base catalyzing the reaction of halogenated hydrocarbons and acid experienced mild conditions, but most of them had a longer reaction time, with complex operations. The reaction of acyl halide esterification had mild reacting conditions, and short reaction time, with high yield, but the use of the acylating agent experienced corrosion and environmental pollution. Witting reaction had mild conditions, short reaction time, and high yield, but the price of triphenylphosphine was high and it was easy to cause pollution to the environment. Malonic acid monoester method had complex reacting steps, and more side reactions. One pot method had mild reaction conditions, simple operations, high yield and the purity was high, while the market price of the 3,4-dihydroxy benzaldehyde was only about 1/3 compared with the price of the caffeic acid, so it indicated the potential scale of chemical synthesis of CAPE.

## 4. Biosynthetic Method

### 4.1. Chlorogenic Acid Hydrolase Catalyzed Synthesis

**Figure 8:**
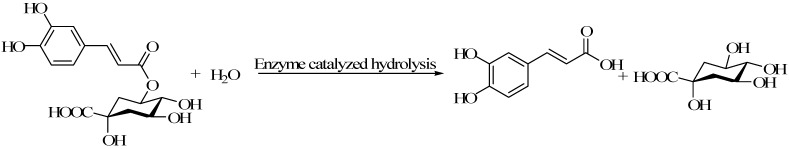
The reactionof enzyme catalytic hydrolysis.

**Figure 9:**
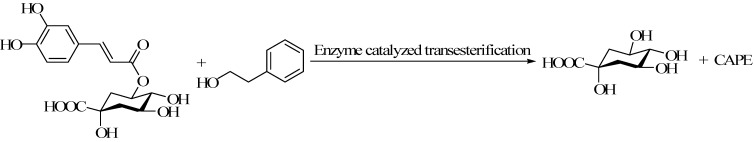
The reactionof enzyme catalytic transesterification.

**Figure 10:**
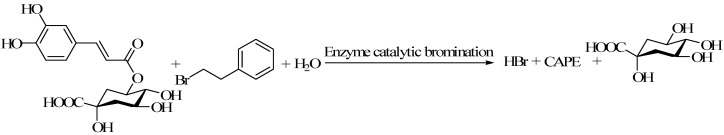
The reactionof enzyme catalytic bromination.

**Figure 11:**
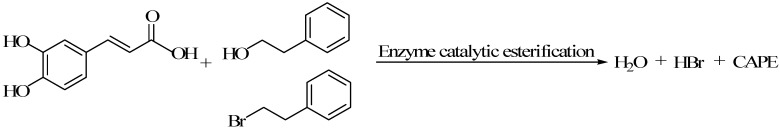
The reactionof enzyme catalytic esterification.

During the study of the reaction system of chlorogenic acid, phenethyl alcohol, β-phenyl ethyl bromide and chlorogenic acid hydrolase, Kishimoto found that chlorogenic acid hydrolase could hydrolyze chlorogenic acid into caffeic acid and quinic acid. Chlorogenic acid hydrolase had the activity of transesterification, and could catalyze chlorogenic acid, benzyl alcohol, and β-phenyl ethyl bromide into CAPE. At the same time, the reaction system (Figure 8, Figure 9, Figure 10 and Figure 11) had also occurred in the substitution reaction of bromine, and catalyzed caffeic acid, β-phenyl ethyl bromide and water into CAPE [62].

During the system, the optimum pH of enzyme catalytic hydrolysis was neutral (pH 6.5), while the optimum pH of the catalytic transesterification and esterification was acid (pH 3–4). The optimum mass fraction of phenethyl alcohol and β-phenyl ethyl bromide was 5%–70%. Under this condition, the enzyme at 40 °C catalytic transesterification reaction of 4 h gave a maximum molar conversion rate of 50%, while the maximum molar catalytic esterification gave a conversion rate of 13%.

### 4.2. Lipase Catalyzed Synthesis

Stevenson [63,64] reported, candida antarctica lipase B (Novozym435) had the scope to generate CAPE by catalytic esterification of caffeic acid and phenethyl alcohol. Accordingly, 0.5 mmol caffeic acid and 0.5 mmol phenethyl alcohol were added into the solvent of tert butyl alcohol (2 mL tert butyl alcohol + 0.2% BHT), and reacted for 500 h. Maximum conversion rate of CAPE was 40%. The esterification reaction could generate water, and the enzymatic reaction was inhibited, so the reaction needed molecular sieve added to remove water.

Widjaja optimized conditions of the generation system to catalyze caffeic acid and phenethyl alcohol into CAPE by Novozym 435 [65]. The results showed that although caffeic acid had low solubility in nonpolar solvents, its conversion rate in nonpolar solvents was much higher than that in polar solvents. When the reacting temperature was 70 °C, caffeic acid had high solubility, and the conversion rate was improved. The optimum molar ratio of caffeic acid and phenethyl alcohol was 1:92. The increase of phenethyl alcohol content could cause the enzyme to be inactivated. The optimum molar ratio of caffeic acid and enzyme was 1:15. The conversion of the esterification reaction within 48 h rate was 100%. Reaction did not need stirring; 50 r min was sufficient to allow the substrate to mix. The reaction without stirring could make the stability of enzyme better, but the conversion rate was usually less than 90%.

The biological enzyme method for the synthesis of CAPE had the advantages of mild conditions, simple operations, low cost, high conversion rate and less damage to the environment, but still had the disadvantage of low optimum molar ratio of caffeic acid and phenethyl alcohol, high solvent consumption, and low efficiency of industrialization. Using mature chemical design and the key technology of reaction-separation coupling were the most important research directions for large scale preparation of CAPE, and to solve the problems of catalytic synthesis of CAPE.

## 5. Synthesis of CAPE Derivatives and Their Structure-Activity Relationships

### 5.1. Two Adjacent Phenolic Hydroxyls Substituted Derivatives

**Figure 12:**
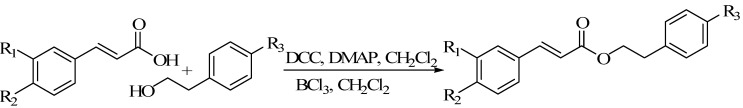
The scheme of synthesis of CAPE derivatives.

**Figure 13:**
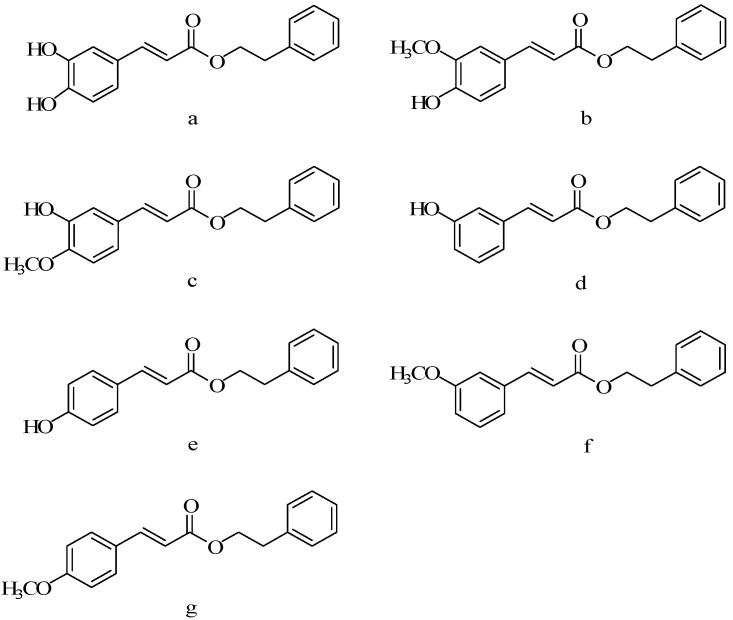
ThestructureofCAPEderivatives.

By using DCC/DMAP method (Figure 12), Ya-Ting Lee synthesized 14 CAPE derivatives (Figure 13) and studied the effects of this series of compounds on oral squamous cell carcinoma, oral epithelial carcinoma cells and normal oral fibroblasts by MTT assay and trypan blue staining method [66]. Experiments showed that compounds **a**–**d** in 5–100 µM concentration range had the cytotoxic effect on oral squamous cell carcinoma and oral epithelial carcinoma cells, but had no effect on normal oral fibroblasts. The IC_50_ of **a**, **b**, **e** on oral epithelial carcinoma cells measured by MTT method were 159.2 ± 7.2, 87.6 ± 6.0, 42.6 ± 4.3 µM. The cytotoxicity of compounds **b** and **c** on oral epithelial carcinoma cells was significantly higher than CAPE. When a hydrogen atom substituted a hydroxyl of the two adjacent phenolic hydroxyl groups (**d**, **e**) or a methoxy group and a hydrogen replaced the two adjacent phenolic hydroxyl groups at the same time (**f**, **g**), anticancer activity was lower than CAPE. Thus, when a hydrogen atom of the two adjacent phenolic hydroxyl groups was substituted by a methoxy group, it was advantageous to enhance anticancer activity.

### 5.2. Benzene Hydrogen Substituted Derivatives

**Figure 14:**
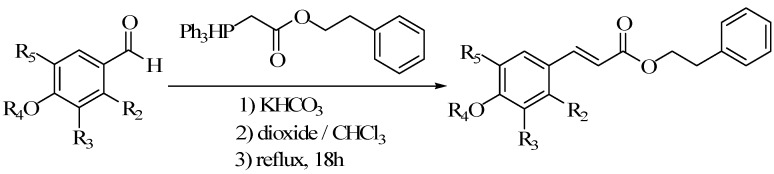
The scheme of fluorine substituted derivatives of CAPE.

**Figure 15:**
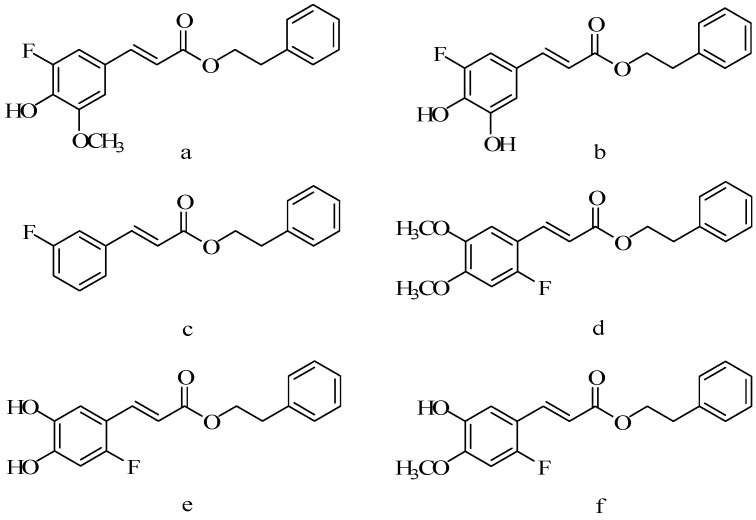
The structure of fluorine substituted derivatives of CAPE.

Some compounds exist “halo effect”, when the halogen was introduced into the structure (Figure 14), its biological activity was enhanced. Xin-yu Wang synthesized a series of CAPE derivatives (Figure 15) substituted with fluorine by witting coupling method, and studied the cytotoxicity of the derivatives and the protective effect of oxidative stress in endothelial cells induced by menadione [67].

Cell protective effects were displayed, and except for compound **b**, all of the derivatives were substituted by fluorine, and these cell protective effects were dose dependent; the cytoprotective role of compound **e** is similar to CAPE. Others were lower than that of CAPE. From the results of cell protective effect, it could be seen that the fluorine substitution on the phenyl ring position was related to activity. The activity of the compounds which fluorine substituted in 2-position was stronger than that in the 3-position. Chun-nian Xia synthesized 20 caffeic acid derivatives and determined its anticancer activity by MTT method [68].

### 5.3. Alkyl Chain Extended Derivatives

**Figure 16:**
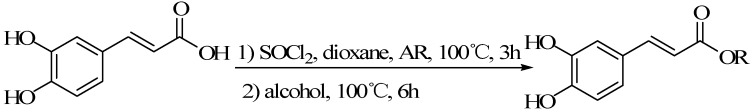
The scheme of elongation of alkyl derivatives of CAPE.

**Figure 17:**
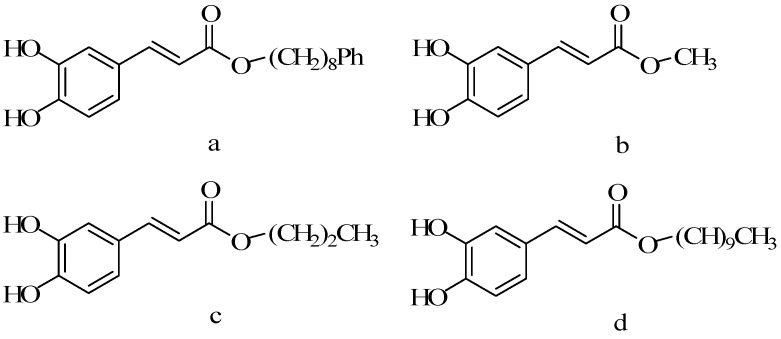
The structure of elongation of alkyl derivatives of CAPE.

Nagaoka T synthesized CAPE and its 20 derivatives (Figure 16 and Figure 17) by the acyl chloride method, using the method of MTT, determining the effect on the rat colon carcinoma cells 26-L5, mouse melanoma cell line B16-BL6, mouse Lewis lung cancer cells LLC, human fibroblast cell HT-1080, human lung cancer cell A549 and human cervical carcinoma cell line HeLa [46]. According to differences in alcohol parts of the structure, all of the CAPE derivatives could be divided into four categories: the first category was the alkyl chain end connected with a benzene ring, the second category was the alkyl chain end connected with styrene, the third category was the alkyl chain end connected with cyclohexyl, and the fourth category was the alkyl chain end. The anticancer activity of the compounds from the first class increased with the increase of the alkyl chain. The activity of compound **a** was the highest. The EC_50_ to 26-LS, B16-BL6 and LLC were 0.09, 0.84 and 1.81 µM. Further increasing carbon chain did not increase the activity. In addition, the compounds of **b**–**d** from the fourth class also showed similar regularity, with the alkyl chain by one carbon atom to 10 carbon atoms increasing, while the EC_50_ decreased. The compounds have been studied inhibiting LPS stimulated J774.1 to produce no activity [69]. The results showed that the compounds from the first class compared with the compounds from third class had stronger activity, proving that benzene ring in the structure of derivatives of CAPE had an important effect on the activity.

### 5.4. Caffeic Amide Derivatives

Ya-Ting Lee synthesized four CAPE derivatives by DCC/DMAP method and studied their effects on oral squamous cell carcinoma, oral epithelial carcinoma cells and normal oral fibroblasts [66] by MTT assay and trypan blue staining method. Experiments showed that the amide derivatives compared with caffeic ester derivatives, the activity was lower than caffeic ester derivatives. Visibly, the oxygen atoms of caffeic ester derivatives had important effects on the activity.

## 6. Conclusions

**Figure 18:**
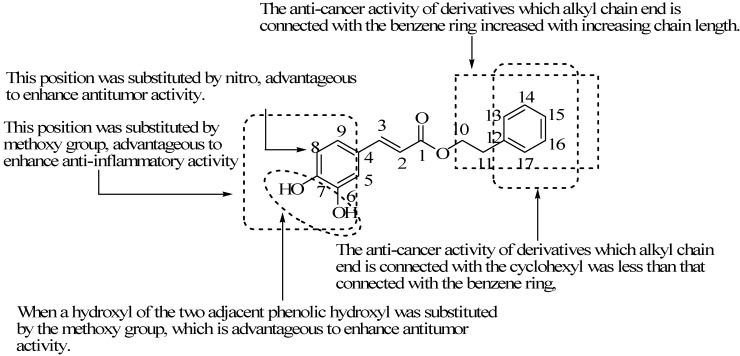
The structure-activity relationship of CAPE and its biological activity.

The main synthetic routes of CAPE are chemical synthesis and biosynthesis, with anti-oxidant, anti-inflammatory, anti-tumor activities. Substituting different positions of the structure of CAPE can cause changes in activities. The structure-activity relationship of CAPE and its biological activities are shown in Figure 18.

When a hydrogen atom substitutes a hydroxyl of the two adjacent phenolic hydroxyls or a methoxy group and a hydrogen atom substitutes the two adjacent phenolic hydroxyls at the same time, the anticancer activity is lower than CAPE. When a hydroxyl of the two adjacent phenolic hydroxyls is substituted by a methoxy group, it is advantageous to enhance anticancer activity. In addition, 7-hydroxyl in CAPE structure is substituted by a methoxy group, advantageous to enhancing anti-inflammatory activity.

Some compounds demonstrate a “halo effect”; when the halogen is introduced to the structure, its biological activity is enhanced. However, the hydroxyl of the CAPE benzene ring is substituted by a halogen atom, its anticancer activity is not significant. However, the hydroxyl on the 8-position is substituted by nitro, advantageous to enhancing anticancer activity.

The alkyl chain length of CAPE can cause the changes of the activities; the anticancer activity of derivatives which the alkyl chain end is connected with in the benzene ring increases with increasing chain length. The anticancer activity of derivatives which the alkyl chain end is connected with in the cyclohexyl is less than that of derivatives which the alkyl chain end is connected with in the benzene ring, showing the benzene ring in the structure of CAPE derivatives plays an important role in the activity.

Therefore, (1) Two adjacent phenol hydroxyls is an important active group of the CAPE, but the substitution of one hydroxyl group can enhance the activity; (2) Hydrogen atoms on the benzene ring are determined by some electron withdrawing groups, which is helpful to increase the activity, such as nitro, halogen; (3) Appropriate extension of the alkyl chain of the alcohol part can cause the activity to increase to a certain extent.

Through extensive study of the structure–activity relationship of CAPE, CAPE derivatives with stronger activities will be designed and synthesized.
